# ICPNet: Advanced Maize Leaf Disease Detection with Multidimensional Attention and Coordinate Depthwise Convolution

**DOI:** 10.3390/plants13162277

**Published:** 2024-08-15

**Authors:** Jin Yang, Wenke Zhu, Guanqi Liu, Weisi Dai, Zhuonong Xu, Li Wan, Guoxiong Zhou

**Affiliations:** 1 College of Electronic Information and Physics, Central South University of Forestry and Technology, Changsha 410004, China; 20223794@csuft.edu.cn (J.Y.); 20233867@csuft.edu.cn (G.L.); 20212894@csuft.edu.cn (W.D.); 20212927@csuft.edu.cn (L.W.); zhougx01@163.com (G.Z.); 2 College of Bangor, Central South University of Forestry and Technology, Changsha 410004, China; 20216175@csuft.edu.cn

**Keywords:** ICPNet, maize leaf disease detection, deep learning

## Abstract

Maize is an important crop, and the detection of maize diseases is critical for ensuring food security and improving agricultural production efficiency. To address the challenges of difficult feature extraction due to the high similarity among maize leaf disease species, the blurring of image edge features, and the susceptibility of maize leaf images to noise during acquisition and transmission, we propose a maize disease detection method based on ICPNet (Integrated multidimensional attention coordinate depthwise convolution PSO (Particle Swarm Optimization)-Integrated lion optimisation algorithm network). Firstly, we introduce a novel attention mechanism called Integrated Multidimensional Attention (IMA), which enhances the stability and responsiveness of the model in detecting small speckled disease features by combining cross-attention and spatial channel reconstruction methods. Secondly, we propose Coordinate Depthwise Convolution (CDC) to enhance the accuracy of feature maps through multi-scale convolutional processing, allowing for better differentiation of the fuzzy edges of maize leaf disease regions. To further optimize model performance, we introduce the PSO-Integrated Lion Optimisation Algorithm (PLOA), which leverages the exploratory stochasticity and annealing mechanism of the particle swarm algorithm to enhance the model’s ability to handle mutation points while maintaining training stability and robustness. The experimental results demonstrate that ICPNet achieved an average accuracy of 88.4% and a precision of 87.3% on the self-constructed dataset. This method effectively extracts the tiny and fuzzy edge features of maize leaf diseases, providing a valuable reference for disease control in large-scale maize production.

## 1. Introduction

As an important food crop, corn occupies a key position in China’s agricultural sector, and the corn industry is not only closely related to people’s food and clothing but can also bring great economic benefits. Apart from being a vital food source for humans, maize is also utilized for producing edible oils, animal feed, maize flour, and other purposes and is extensively employed as an industrial raw material [1]. However, maize diseases are a major threat to agricultural productivity and can cause significant economic losses [2]. Currently, the way most growers determine the disease category of a crop is still through visual observation and identification by experience. However, with the increasing complexity of disease situations leading to frequent bias in visual observation [3], growers are still struggling to cope with major financial setbacks caused mainly by the prevalence and impact of various maize diseases [4]. Prompt detection and precise identification of different maize disease types are crucial for effective disease management.

With the continuous development and innovation of computer vision technology in the field of crop disease recognition, scientists at home and abroad have carried out lots of work in the field of crop disease image recognition based on computer vision technology and achieved some important results. In the early days, the detection of corn leaf diseases was primarily based on traditional image analysis techniques. Ref. [5] and machine learning [6]. These techniques usually include image preprocessing, feature extraction, feature selection, and classification. However, these methods have some obvious drawbacks. Firstly, manual feature extraction relies on domain knowledge and is a complex process that makes it difficult to capture high-level features and complex patterns in images. In addition, the accuracy and robustness of these methods are usually limited by the effectiveness of feature extraction and the performance of the classifiers, which perform poorly, especially in the presence of complex backgrounds and changing lighting conditions.

In recent years, a number of automatic and convenient methods for maize disease identification have been explored. Some researchers have focused on deep learning techniques, which are considered to have great potential for solving vision-based maize disease identification problems. Ishak Pacal [7] used a lightweight multi-axis vision transformer model, which, by integrating different datasets and employing an advanced Squeeze-and-Excitation (SE) module, significantly improved the accuracy and inference speed, achieving a high recognition accuracy of 99.24%. Although the MaxViT model performs well under experimental conditions, its generalisation ability may be limited by the diversity and representativeness of the training data, making the model’s effectiveness in different agricultural environments and disease types vary. Fang et al. [8] proposed a novel network architecture called HCA-MFFNet, which utilises the Hard Coordinated Attention (HCA) mechanism that adjusts at various spatial scales to derive features from corn leaf pictures, thereby dropping interference from complicated backgrounds. Additionally, the model successfully reduces the number of network parameters by substituting traditional convolutional layers with deep-separable convolutional layers. Despite demonstrating an average recognition accuracy of up to 97.75% in complex environments, there are still limitations in terms of network parameters and training speed. Li et al. [9] developed a deep neural network (MDCDenseNet) combining a multi-expansion module and a convolutional block attention mechanism (CBAM) with an assisted classification generative adversarial network and migration learning to expand the dataset and pre-training, which improved the recognition accuracy to 98.84%. However, when processing image data with complex backgrounds, the model may still face problems of insufficient generalisation ability and slow processing speed. Sun et al. [10] proposed the CASF-MNet network model, which effectively improves the recognition accuracy of the colour and texture features of lesions by introducing the Hue, Saturation, and Vibrance (HSV) colour space and the Cross Attention Mechanism (CAM), showing higher performance. However, the model still exhibits some limitations in dealing with diseased leaves with complex colour and texture features and lacks diversity in training data. Zhang et al. [11] proposed a two-stage classification model called MaizePestNet, which utilises the Grad-CAM algorithm and knowledge distillation strategy to accurately identify pests in maize fields. This study enhanced the identification precision and efficiency of the module, significantly decreased the size of the module, and developed a real-time online identification system based on this model. Nevertheless, there are still limitations in the generalisation ability of the model when dealing with complex backgrounds and the higher computational resources required to train this model.

Although existing studies provide a significant reference value for maize disease recognition in the agricultural field, there remain deficiencies in data representation. These studies mainly rely on public datasets and a small amount of collected data, which often feature overly simplistic image backgrounds and a limited number of training samples, thereby compromising the accuracy of disease recognition in complex backgrounds. The primary problems faced in this study include: (1) The high similarity between maize leaf disease species and the subtle symptoms of some diseases make feature extraction more difficult, and the neural network is prone to disturbances by small and numerous disease spots. This requires the network model to extract finer features to improve recognition accuracy. (2) In practical applications, the backgrounds of corn leaf photos are complex, and the collection environment is variable. The image edge features are often blurred, which affects the network’s recognition accuracy. (3) Corn leaf images are susceptible to noise during the collection and transmission process, resulting in blurred image features that further impact recognition accuracy.

Aiming at the above problems, some studies have provided potential solutions. To address the issue of feature extraction difficulties due to the high similarity among maize leaf disease species, Prasher et al. [12] designed a classifier utilising a CNN model that integrates both SGD and Adam optimisers to identify critical features from a rice leaf image dataset and classify different types of rice disease. Their evaluation demonstrated that the DenseNet201 model when paired with the SGD optimiser, reached an accuracy of up to 95%. On the other hand, Jin et al. [13] addressed the issue by enhancing texture information retention through the use of convolutional integration with residual blocks and the recombination (regr) method, along with the concatenation (concat) method in the generator. These techniques have shown potential for enhancing the accuracy and robustness of disease recognition models, particularly in complex environments.

In order to reduce the interference of image edge feature blurring on network recognition, Li et al. [14] developed an LMBRNet-based method for tomato leaf disorder recognition. They constructed the Comprehensive Grouped Differential Residuals (CGDR) and utilised its multi-branch structure to capture diverse feature information about the disease from different dimensions and sensory domains. Similarly, Deng et al. [15] developed an accurate image-based segmentation technique, MC-UNet, for tomato leaf disorders, which uses the extrusion excitation model to highlight the border feature characteristics of tomato disorders. These approaches have demonstrated the potential to enhance the accuracy of disease recognition by effectively addressing the issue of blurred image edge features.

To address the problem of corn leaf images being susceptible to noise during acquisition and transmission, Chen et al. [16] used the Binary Wavelet Transform in combination with Retinex (BWTR) to denoise and improve pictures. This approach effectively removes noise points and edge points while retaining key texture information. The denoised images were then recognised using a two-channel residual attention network (B-ARNet), achieving an overall detection accuracy of about 89%. Yeswanth et al. [5] introduced an innovative adaptive scale feature extraction super-resolution network (ASFESRN) aimed at detecting maize leaf diseases. ASFESRN incorporates a distinctive S-specific feature decomposition and integration block (FDIB) along with Adaptive Scale Group Convolutional Network (ASGCN) modules, which are specifically designed to enhance the resolution of low-quality field images. This technique reduces the impact of noise on image quality, thereby enhancing the accuracy of disease detection.

The major contributions of this paper are as follows:

(1) To enable the image classification network to capture detailed features of maize leaf diseases, we created a precisely annotated dataset that includes both dongrove and spot diseases.

(2) We propose a novel attention mechanism called Integrated Multidimensional Attention (IMA). This module combines cross-attention and spatial channel reconstruction methods by first using cross-attention for feature extraction, followed by feature weight updating using spatial and channel reconstruction modules. It adjusts the weights based on the cross-information of the features to respond more efficiently to critical changes while ensuring the stability of the model in recognising tiny, speckled disease features.

(3) A novel Coordinate Depthwise Convolution (CDC) is proposed, which improves the quality of the feature map through spatial and channel reconstruction modules. Firstly, the feature map is segmented along horizontal and vertical directions and then processed by two multi-scale convolutions, respectively, where the horizontal multi-scale convolution filters important features and reduces background noise. The vertically orientated convolution optimises the channel information and maintains focus on key features. The combination of these two steps enables the module to improve its ability to distinguish the blurred edges of the corn leaf spot region.

(4) We present a novel optimisation algorithm called the PSO-Integrated Lion Optimisation Algorithm (PLOA). This algorithm merges the exploratory randomness of the particle swarm optimisation with the incremental constraints of the annealing mechanism, designed to prevent the module from getting trapped in local optima. By gradually restricting the exploration range, PLOA enhances the model’s ability to identify maize disease features while maintaining training stability and model robustness.

(5) The ICPNet introduced in this study achieved an average accuracy of 88.4% and a precision of 87.3% on the custom-built dataset. This approach effectively extracts the minute and blurred edges of maize leaf disease features. Overall, it effectively identifies maize leaf diseases and provides valuable insights for managing diseases in large-scale maize cultivation.

## 2. Materials and Methods

### 2.1. Data Acquisition

Maize disease image recognition and classification, from the optimisation and debugging of the algorithm to the evaluation of performance, require indispensable support from a robust dataset. Therefore, to ensure sufficient training data for image recognition and classification, we established a corn disease dataset. This dataset was primarily screened from the public dataset available on the CSDN platform.

Considering the validity of the input images, all images were classified into eight categories: Blight, Cercospora, Gray Spot, Fall Armyworm, Herbicide Burn, Rust, Zinc Deficiency, and Healthy Leaf. The entire dataset contains 4107 images, divided into 3286 to train and 821 to validate. These images are stored in both .jpg and .png formats. Table 1 provides a summary of the maize disease dataset categories, detailing each category and its proportion within the dataset.

### 2.2. Method

#### 2.2.1. Integrated Multidimensional Attention (IMA)

Since the neural network is susceptible to the effects brought about by the small and numerous spots during the maize pest classification process, the introduction of attention in the model is a necessary step to improve the accuracy of classification. Attention mechanisms are extensively utilised in various neural net architectures, including convolutional neural networks [17], recurrent neural networks [18], and transformers [19], which are very efficient in processing image data, time series data, and natural language data. However, the current attention mechanisms cannot adequately understand the relationships and dependencies between spot elements when dealing with diverse and tiny spot data, resulting in poor generalisation. The earlier SE [20] model enhanced the attention and expressiveness of different channel features by modelling the importance of each feature channel separately. This approach not only improved the network’s performance but also effectively reduced redundant information between channels, enhancing the network’s stability. Subsequent models like CBAM [21] added spatial attention to SE to enhance the expression of features both spatially and channel-wise, effectively extracting spatial patterns and variations in the image. However, for the task of maize disease classification with diverse spots, using only channel attention or spatial attention often yields unsatisfactory results due to the lack of localisation or single feature information. Although CBAM’s integrated information approach addresses some of these issues, it still lacks interaction and dependency between elements. For this reason, we propose Integrated Multidimensional Attention (IMA).

**Figure 1:**
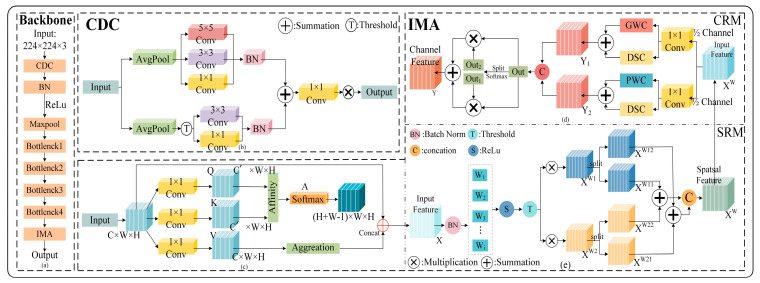
Structure diagram of ICPNet. (**a**) Backbone network structure; (**b**) CDC structure; (**c**–**e**) IMA structure.

Considering the complexity of tiny speckled lesions, we use IMA as the attention mechanism of the network, as shown in Figure 1. The IMA module consists of two main parts.

(1) Feature Extraction:

Assuming the input maize disease feature map has dimensions $X\in R^{C\times H\times W}$, where C represents the number of input channels and H and W indicate the height and width of the input feature map, respectively. After passing through three 1 × 1 convolutional layers, it is filtered to generate three feature maps: Q, K, and V. Q and K undergo the affinity operation, and V undergoes the aggregation operation, as follows:

① The Affinity operation is as follows:

(1) $$d\in Q_{u}\Omega_{i,u}^{T}$$

where $d_{i,u}$∈D is the feature map, $Q_{u}$ and $\Omega_{i,u}$ are intermediate feature maps, and $Q\in R^{C^{\prime }}$ is the vector derived from Q at each position u in the spatial dimension, $i=\left[1,\cdots \left|\Omega_{u}\right|\right]$. $\Omega_{i,u}\in R^{\prime }$ is the i-th element of the feature vector extracted from K that is in the same row or column as position u. $D\in R^{\left(H+W-1\right)\times W\times H}$, H and W are the height and width of the input feature map, respectively; and C’ is the number of output channels. The attention map $A\in R^{\left(H+W-1\right)\times W\times H}$ is generated by the affinity operation and is finally normalised by the softmax operation. The affinity operation is used to calculate the similarity between each position and other positions in the feature map. The cross-attention module reduces computational complexity by utilising the features on the crossover paths.

② Aggregation operation is as follows:

Let the input feature map be $X\in R^{C\times H\times W}$. A linear transformation is performed on X to obtain $X\in R^{C\times H\times W}$, where C″ is the number of channels after dimensionality reduction. A reshape operation is then performed on *g*(*X*) to obtain, where *N* = *W* × *H* is the amount of pixels. Finally, the attention weight matrix is multiplied by the feature matrix g(X) to obtain the final feature matrix.

(2) Feature Weight Update

To update the feature weights, we use the Spatial Reconstruction Module (SRM) and the Channel Reconstruction Module (CRM) of SCconv [22]. Firstly, spatial reconstruction is performed on the initially processed feature maps. This is operated as follows:

After inputting the feature into SRM, the following stages are outlined below: For the input corn feature map, better feature fusion is achieved by separating and reconstructing its operation. First, a batch normalisation layer (BN) is designed to normalise it and generate the intermediate value *X_out_*. Then, *X_out_* is mapped by a sigmoid function to produce a weight score W from 0 to 1. The step is crucial for the following gating operation, which detects significant features according to a defined threshold. During the procedure, the model distinguishes between features that are crucial for detecting diseased areas on maize leaves and those that are less important. Two new weight values, *W_1_* and *W_2_*, are determined by comparing W with a set gating threshold gate_threshold, where *W_1_* is the value for the more meaningful maize leaf disease features. (significant features), *W_2_* represents the weight for the less informative features (less important features). Finally, the two weights *W_1_* and *W_2_* are multiplied by the original feature X and split to generate four different weights: *X^W11^*, *X^W12^*, *X^W21^*, and *X^W22^*. These are cross-constructed to generate the new output *X^W^*, which integrates both meaningful and non-meaningful parts. The entire procedure of separation and reconstitution can be described as follows:

(2) $$X_{out}=\gamma \left(\frac{X-\mu_{B}}{\sqrt{\sigma_{B}^{2}+\epsilon }}\right)+\beta$$

(3) $$W=Gate\left(sigmoid\left(BN\left(X_{out}\right)\right)\right)$$

(4) $$\{\begin{array}{l} X^{W_{1}}=X⊗W_{1}\\ X^{W_{2}}=X⊗W_{2}\\ X^{W}=\left(X^{W_{11}}⊕X^{W_{22}}\right)⊕\left(X^{W_{21}}⊕X^{W_{12}}\right) \end{array}$$

The pooled features are processed by SRM (Selective Feature Enhancement Module) to distinguish between important and unimportant features in maize leaf diseases.

This enables the system to concentrate better on features that are relevant to the recognition and segmentation of maize and to disregard extraneous and noisy background elements. This characteristic of separation processing significantly improves feature extraction when dealing with complex agricultural scenarios. Therefore, the combination of ⊕ and ⊗ operations play a key role in feature processing and further enhances the detection and segmentation capabilities of the network.

Channel reconstruction is first performed on the initially processed feature maps as follows:

Initially, the input feature map for maize leaf disease is split into 2 parts, each with an equal number of channels, denoted C. The features corresponding to half of the channels (1/2 C) are directed to the upper feature layer (UP), while the remaining features (the other 1/2 C) are directed to the lower feature layer (LOW). The channels in both the upper and lower features are then compressed using two parallel 1 × 1 convolutions (1 × 1 Conv) to increase computational efficiency. This process produces two feature maps, $X_{UP}^{W}$ and $X_{LOW}^{W}$. The upper feature, $X_{UP}^{W}$, captures broader contextual details, such as the whole shape of the leaf, while the bottom function, $X_{LOW}^{W}$, concentration on detailed aspects of leaf disorders, like specks and discoloured areas.

To process the top and bottom features more efficiently, we adopt advanced convolution techniques such as Grouped Convolution (GWC), Pointwise Convolution (PWC) and Depthwise Separable Convolution (DSC) in place of the traditional complicated k×k convolution, thus decreasing the computational expense.DSC splits the convolution process into deep convolution and spot convolution, requiring only 1/C of the initial computation, making it more efficient. PWC mitigates information loss during convolution and facilitates information exchange between channels. GWC improves overall computational efficiency by grouping channels and using different convolution kernels for each part.

Specifically, for the upper-layer feature $X_{UP}^{W}$, we apply a 3 × 3 Grouped Convolution (GWC) and Depthwise Separable Convolution (DSC) to thoroughly extract general and local features of maize diseases at a lower computational expense, resulting in the upper-layer feature map $Y_{1}^{W}$. At the other branch, we process $X_{LOW}^{W}$ with a DSC and a more efficient 1 × 1 PWC to capture features with shallow hidden disease location information. The resulting lower feature map $Y_{2}^{W}$ complements the upper features. The specific manipulations of 2 branches are as follows:

(5) $$\{\begin{array}{l} Y_{1}^{W}=G\left(X_{UP}^{W}\right)+D\left(X_{UP}^{W}\right)\\ Y_{2}^{W}=P\left(X_{LOW}^{W}\right)+D\left(X_{LOW}^{W}\right) \end{array}$$

Through this layered processing strategy, the CRM module effectively improves the accuracy and efficiency of feature extraction and further optimises the detection and classification performance of maize leaf disorders.

Lastly, the top feature map $Y_{1}^{W}$ and bottom feature map $Y_{2}^{W}$ are merged along the channel direction to create the merged feature map. A softmax performance is then applied to the merged feature map to produce the attention weights. The attention weight tensor is divided into 2 parts along the channel direction and multiplied by the fusion feature map. Lastly, the outcomes of these 2 multiplications are added together to produce the end feature map $Y_{W}$.

Next, $X_{H}^{W}$ and Y obtained by SRM and CRM are concatenated along the channel dimension to form y. This concatenated feature is then downscaled and transformed using a 1 × 1 conv, BN layer and sigmoid activity function. It is then split into $y_{h}$ and $y_{w}$ in the space dimension and transformed into an attention map by two parallel convolution operations. Finally, it undergoes sigmoid processing and is multiplied by the principal component of the initial feature map identity to produce the last maize leaf disease feature map. The sequence of operations after generating $X_{H}^{W}$ and Y are as follows:

(6) $$Y=Concat\left(X_{H},Y\right)$$

(7) y=sigmoid(BN(1×1Conv(Y)))

(8) $$\{\begin{array}{l} a_{h}=sigmoid\left(1\times 1Conv\left(y\right)\right)\\ a=sigmoid\left(1\times 1Conv\left(y\right)\right) \end{array}$$

(9) $$Out=identity\times a_{w}\times a_{h}$$

In Section 3.2.1 of this paper, by specifically analysing the effect of the IMA module, we can see that its comparison experiments show a significant performance improvement over the baseline model. This illustrates that the proposed enhancement is both theoretically robust and superior in practical applications.

#### 2.2.2. Coordinate Depthwise Convolution (CDC)

The initial convolution operation in the ResNeXt-50 model can effectively capture the fuzzy edge features in maize pest data, improving the model’s capacity to detect edge features. Anyway, the single 7 × 7 convolution’s feature extraction capability is limited, which cannot effectively handle numerous fuzzy features, and the convolution kernel is too large, increasing the computational complexity in the edge feature extraction process. Therefore, to avoid the problem of reduced model generalisation ability when inputting edge fuzzy features, this paper proposes a Coordinate Depthwise Convolution (CDC) with the structure shown in Figure 1b.

While the common 3 × 3 convolution has been able to capture edge information in conventional maize disease datasets, the mapping range of the fixed convolutional layers is limited, which does not capture the overall information in large-scale datasets and affects classification accuracy. In addition, a fixed convolutional filter considers all sampling points in the disease data equally, which can limit the model’s ability to express fuzzy edge features. To address this issue, we constructed the convolutional layer using CDC instead of the 7 × 7 convolution in the preliminary processing section. First, we borrow the core idea of Coordinate Attention (CA) [23]. We use two parallel adaptive pooling layers to decompose their feature maps into two dimensions, vertical and horizontal, for feature extraction separately. This operation enables the module to focus on which changes are more significant at different coordinate points of the feature maps, which is particularly important for understanding edge features.

Due to the fuzzy edges characteristic of maize pest data, important information may be hidden in different dimensions in the classification of pest images. In response to this, we designed a multi-scale convolutional structure to capture features in the vertical direction, consisting of three parallel branches using convolutional operations with kernel sizes of 1 × 1, 3 × 3, and 5 × 5, respectively. Each branch is equipped with a normalisation layer to stabilise network training and prevent divergence. The feature maps from the three branches are ultimately combined through concatenation. In the horizontal dimension, traditional convolution processes all features as inputs, which not only increases computational effort but also reduces the generalisation ability of the network. To solve this problem, we introduce a gating mechanism to classify features into important and unimportant features. For important features, we use a 3 × 3 convolutional kernel to extract edge information more comprehensively, while for unimportant features, we use a 1 × 1 convolutional kernel to save computational resources. Finally, the output feature map is obtained after processing by convolution, normalisation, and the Sigmoid activation function.

Specifically, we design a structure that combines multi-scale convolution and gated convolution to cope with edge-ambiguous features in maize pest and disease data. First, the adaptive pooling layer decomposes the input feature map into vertical and horizontal dimensions. In the vertical dimension, we extract features using multi-scale convolution, employing different-sized convolution kernels and normalisation for each branch. In the horizontal dimension, we introduce a gating mechanism to optimise computational resources by applying different-sized convolutions for important and unimportant features, respectively. Ultimately, the extracted feature maps are concatenated and fed into the classification network to ensure efficient and accurate feature extraction, thereby improving the performance of the classification task. Through this combination of multi-scale convolution and gated convolution, we are able to perform accurate feature extraction of edge-ambiguous feature maps, providing rich and accurate feature information for the subsequent classification task. This approach not only improves the computational efficiency of the network but also enhances its robustness and accuracy in dealing with complex and variable features. Experiments on CDC are explained in Section 3.2.2.

#### 2.2.3. PSO-Integrated Lion Optimisation Algorithm (PLOA)

Due to increased stability during model training and improved classification accuracy, we propose the PSO-Integrated Lion Optimisation Algorithm (PLOA) to stabilise the training of ICPNet classification models. The dynamic changes of parameters (e.g., learning rate, momentum coefficient) during training affect the robustness and accuracy of the model. However, the traditional parameter tuning strategy may not be flexible enough in some cases, particularly when facing complex features such as multiple maize disease classes. Therefore, we chose the more robust Lion algorithm [24] as the basis for proposing PLOA for parameter optimisation. At the beginning of training, the PLOA optimiser is launched with a high initial learning rate to accelerate the early global search process. As training proceeds, the learning ratio is gradually reduced by simulating the annealing mechanism [25] to achieve stable convergence. This adaptive strategy not only accelerates the model’s progress towards the global best result but also fine-tunes the parameters in the later stages of training to avoid oscillations or deviations that can be caused by overly large learning steps. In addition, to increase the parameter exploration space of the optimiser, PLOA introduces an advanced particle swarm algorithm (PSO) [26], which searches for the optimal solution by simulating the collaboration and shared information mechanism of the particle population. In PSO, each particle moves in the search space and adapts its location according to its own experiences and the experiences of the population in order to reach the global optimal solution. By introducing stochastic perturbations, PSO enhances the exploration capability of the model and prevents it from falling into local minima.

After training begins, the PLOA optimisation algorithm first initialises the parameters and verifies the learning rate and the coefficients used to get the running mean of the gradient and the square of the gradient. To prevent overfitting, a weight decay operation is first performed to penalise large weight values and encourage the model to learn smaller weights. Then a momentum update operation is performed to accelerate learning and reduce parameter oscillations by calculating new exponential moving averages that are applied to real-time parameter updates. The above process is represented as follows:

(10) $$l_{u}=\beta_{1}\cdot e_{a}+\left(1-\beta_{1}\right)\cdot g$$

where $l_{u}$ is the momentum update of Lion’s algorithm, $\beta_{1}$ is the momentum coefficient, $e_{a}$ A is the exponentially moving average of the slope, while g is the actual slope. Then, PSO updates are performed on the parameters, first on the velocity, in the following process:

(11) $$v_{i}^{k+1}=\alpha \cdot i+\beta \cdot p_{a}+r\cdot g_{a}$$

(12) i=randn(size)·T

$v_{i}^{k+1}$ is the velocity of particle i at generation k + 1, α is the inertia weight that controls the effect of the particle’s previous velocity on its current velocity, β is the cognitive coefficient that controls how much the particle follows its own best position, r is the social coefficient that controls how much the particle follows its global best position and rand (size) is a random number that follows a standard normal distribution.

Then the individual attraction term is calculated for each particle, which attracts the particle to its own historical best position:

(13) $$p_{a}=randn\left(size\right)\cdot \left(p_{i}-x_{i}^{k}\right)\cdot T$$

where p_i_ is the historical best position of particle i, and x_i_^k^ is the position of particle i in the kth generation. The historical best position of the whole population is then used to guide the particle movement, that is, the global attraction term is calculated:

(14) $$g_{a}=randn\left(size\right)\cdot \left(g-x_{i}^{k}\right)\cdot T$$

At last, recognising that continuous use of the Sparrow algorithm during the learning phase can lead to increased computational demands and reduced training efficiency, we incorporated an annealing mechanism. This concept is inspired by the simulated annealing method in materials science, where the temperature of a material is gradually reduced to minimise internal defects and achieve a more stable condition. At the beginning of the training, the annealing temperature T is set higher, leading to a larger magnitude of random perturbations. This helps the parameters be searched over a wider range, thus avoiding prematurely falling into a local optimum. As training progresses, the annealing temperature is gradually lowered, and the perturbation magnitude decreases. This smooth transition helps to shift the search focus from global exploration to local exploitation. In the later stages of training, the annealing temperature drops to a lower level, making the perturbation magnitude very small. This helps in fine-tuning the parameters and avoiding large step-size updates that lead to deviations from the optimal solution. T is varied as follows:

(15) $$T=\max\left(T_{\min},T\cdot a_{r}\right)$$

Finally, the parameters and momentum averages are updated, as follows:

(16) $$x_{i}^{k+1}=x_{i}^{k}+c_{u}$$

(17) $$c_{u}=l_{u}+v_{i}^{k+1}$$

(18) $$e_{a}=\beta_{2}\cdot e_{a}+\left(1-\beta_{2}\right)\cdot grad$$

where $c_{u}$ is the total update for Lion optimisation and PSO update and $\beta_{2}$ is the momentum decay factor.

PLOA’s adaptive gradient tuning capability allows the algorithm to be optimized for different parameters and different training phases, effectively addressing the potential drawbacks of parameter optimisation that may lead to low accuracy and slow convergence of the model in the segmentation phase. PLOA improves the stability of the training of the ICPNet model and enhances the accuracy and robustness in classifying a wide range of complex maize images. Experiments on PLOA are explained in Section 3.2.3.

## 3. Results and Analysis

### 3.1. Experimental Environments and Training Details

To ensure consistency in results, entire experiments in this test were performed using the same hardware and software configurations. The major hardware devices used for the experiments include an NVIDIA GeForce RTX 3090 (24GB) and a 24 vCPU AMD EPYC 7642 48-core processor. While the versions of Python, CUDA and CUDNN do not affect the results of the experiments, they must be compatible with the software and hardware used. We deployed ICPNet on Pytorch 1.10.0 and Pycharm Community Edition 2024.1.6 with the specific experimental parameter settings shown in Table 2.

**Figure 2:**
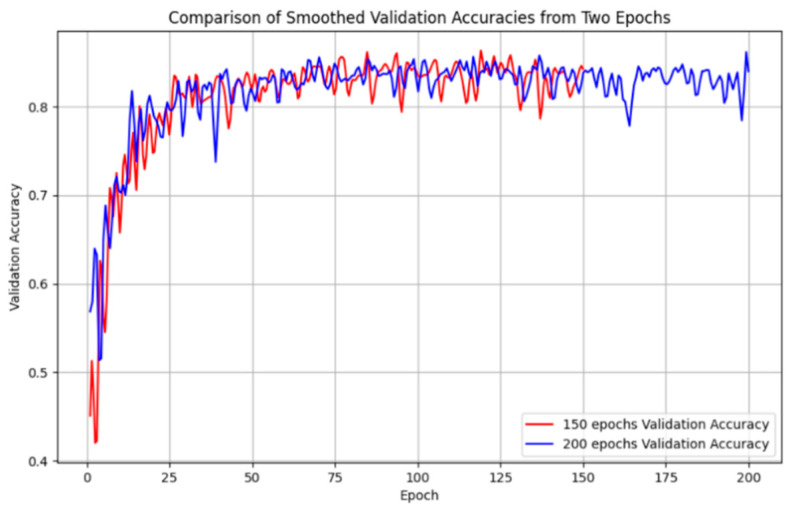
Loss transformation under different iterations.

Based on the ResNeXt-50 source code, we compared the accuracy of the process with 150 and 200 rounds of training. The accuracy figure in the training process is shown in Figure 2. We found that the verification accuracy fluctuates between 0.80 and 0.85 once the training reaches between 75 and 100 rounds. However, to ensure sufficient training, we chose 150 rounds as the number of training rounds for this model.

To analyse the classification outcome, we use Accuracy, Precision, Recall, and F1-score as evaluation metrics. Accuracy denotes the proportion of samples correctly predicted by the model out of all the samples. Precision is the proportion of correctly predicted positive samples to the overall number of samples predicted to be positive. Recall represents the proportion of actual positive class samples that are correctly identified by the model. The F1-score, a harmonic mean of Precision and Recall, provides a comprehensive evaluation of the model’s performance. The equations for calculating these four metrics are below:

(19) $$Precision_{i}=\frac{TP_{i}}{TP_{i}+FP_{i}}$$

(20) $$Recall_{i}=\frac{TP_{i}}{TP_{i}+FN_{i}}$$

(21) $$F1-score_{i}=2\times \frac{precision_{i}\cdot Recall_{i}}{precision_{i}+Recall_{i}}$$

(22) $$Acc=\frac{1}{n_{c1}}\sum_{i=1}^{n_{c1}}\frac{n_{ij}}{n_{i}}$$

where TP, FP and FN represent true positive, false positive and false negative measurements separately. The parameter i is the category index, N is the sum of the number of instances, and n is the number of different categories. The variables TP indicate the number of correctly predicted instances for the first categories, where j is the prediction result and i is the category index. This variable counts the amount of correctly identified initial categories.

### 3.2. Experiments on Module Effectiveness

#### 3.2.1. Validity of IMA

In this paper, we employ IMA as the attention mechanism to improve the model’s stability when dealing with small and numerous spots. To test its validity, we contrast it with several commonly used attentional schemes, including SE, CBAM, CA, ECA [27], and TA [28]. The experimental results are shown in Table 3.

The experimental results suggest that, in addition to SE and TA, other attentional mechanisms can remove interference and enhance classification accuracy to a degree, although the improvement is not substantial. The SE attention mechanism, in particular, overlooks spatial and positional information by focussing solely on the channel dimension, resulting in a decline in classification accuracy. On the other hand, TA becomes more complex in model structure due to the introduction of multiple AttentionGate modules. This complexity makes the model more difficult to train during the optimisation process and requires more hyperparameter tuning and training techniques, resulting in lower accuracy. While CBAM includes spatial attention, it remains inadequate for capturing full contextual information. Taken together, SFAM is more effective and more suitable for the task of classifying maize diseases.

#### 3.2.2. Validity of CDC

In this paper, we use CDC as the initial 7 × 7 convolutional layer of the framework to enhance the robustness of the framework for fuzzy edge features. To confirm its effectiveness, we compare several recent convolutions, such as Ghost Conv [29], Involution [30], and Atrous Conv [31]. The experimental findings are presented in Table 4.

From the experimental results, Ghost Convolution can deal with the interference of fuzzy edges on the classification to a certain extent, but the effect is not significant. Due to the gridding effect and extra inner product operation in Atrous Convolution and Involution, there is a lack of correlation between the convolution results, so the classification effect is not improved enough. Additionally, 7 × 7 convolution requires more computational and parametric quantities, which increases the complexity of pattern training and negatively impacts the classification results. Taken together, CDC is more effective and more suitable for the classification task of maize diseases.

#### 3.2.3. Validity of PLOA

**Figure 3:**
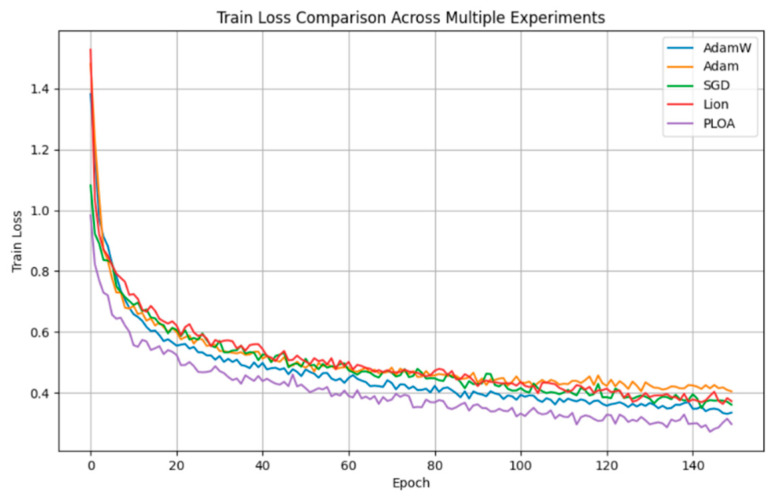
Training loss curves for different optimisers.

In this paper, we use PLOA to stabilise the training of the model. In order to confirm its effectiveness, we compare several popular optimisation algorithms such as Adam [32], SGD [33], AdamW [34] and Lion. The experimental outcomes are presented in Table 5 and Figure 3.

The experimental results demonstrate that PLOA outperforms other optimisation algorithms on our custom training set. Firstly, PLOA shows a significantly lower loss curve, indicating superior convergence behaviour on the training set. In particular, PLOA’s loss value decreases rapidly at the beginning of training, suggesting that it learns more rapidly and finds the best solution quickly. PLOA also leads in crucial performance metrics, having the highest Accuracy, Precision, and Recall, further confirming its precision in image classification tasks. In conclusion, PLOA effectively stabilises the training of the model through its progressive stochastic search approach, guaranteeing stable convergence and attaining the minimal loss profile. This method enhances classification accuracy, enabling PLOA to surpass currently common optimiser algorithms in relation to efficiency.

#### 3.2.4. Ablation Experiments

To validate the efficacy of the ResNeXt-50-based technique described in the study, we performed ablation experiments on ICPNet. The outcomes are displayed in Table 6. We applied the control variable method to sequentially integrate IMA, CDC, and PLOA, performing 8 sets of ablation experiments with these modules in ICPNet. The findings indicate that our CDC module notably boosts the network’s classification performance. When added alone, CDC can improve Accuracy by 3.2% and Precision by 2.6%, proving that CDC is able to perform accurate feature extraction on the feature map at multiple scales, thus improving the classification accuracy of diseased maize leaves. Additionally, we optimised the network using IMA and PLOA. Although the improvements in Accuracy and Precision are relatively modest, with increases of 2.9% and 2.4% for Accuracy and 2.9% and 1.8% for Precision, respectively, they are still effective. In summary, each module of ICPNet positively impacts the model’s classification Accuracy and Precision, confirming the effectiveness of our proposed IMA, CDC, and PLOA.

### 3.3. Comparison with State-of-the-Art Methods

**Figure 4:**
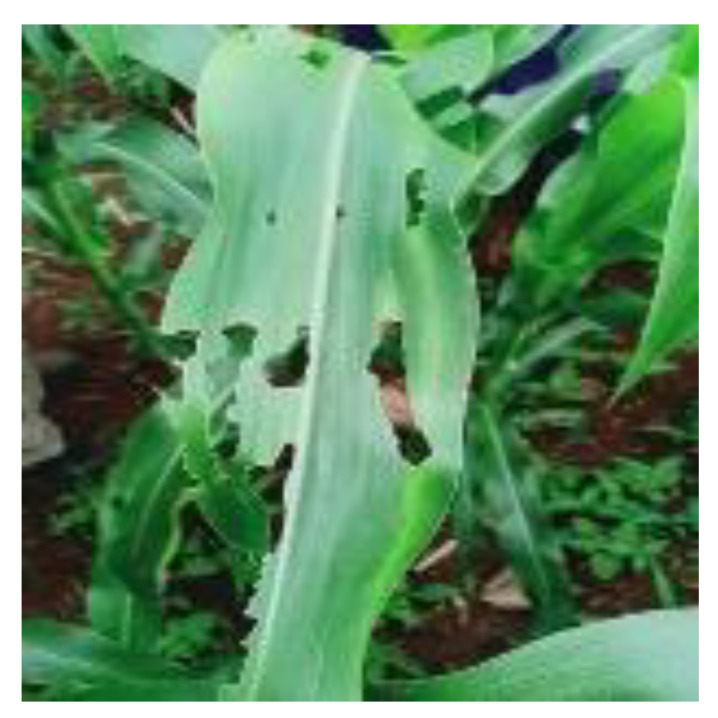
Confusion matrix identification result.

We contrasted ICPNet with traditional Convolutional Neural Networks and the latest state-of-the-art models, including GoogLeNet, ResNet-50, DenseNet-121, MobileNetV2, ResNeXt-50, RegNetY-400MF, CMT, and ICAI-V4. We standardised the hyperparameters to Batch_size = 32, learning rate = 0.001, and Epoch = 150 for our experimental comparisons. The confusion matrix (refer to Figure 4) illustrates the individual model’s performance in disease detection, showing high accuracy across all categories. Table 7 presents a comparison of the different classification metrics. It can be observed that our ICPNet model has significant advantages over traditional and recent state-of-the-art networks in the field of maize disease recognition.

Table 7 shows that ICPNet has higher recall indicators for Healthy, Bacterial Spot, and Mosaic Virus than for Anthracnose and Phytophthora Blight. This improvement is due to the IMA module in the base network, which effectively captures interactions and dependencies between disease elements and enhances feature extraction, particularly for small-scale diseases. Additionally, ICPNet attains an average accuracy of 88.46%, which is the highest among all compared network models, fully proving the feasibility of ICPNet. Figure 4 also demonstrates that ICPNet achieves the greatest recognition rate and the most correct identifications.

In the complexity matrix presented in Figure 4, the amount in each cell represents the number of classification results for each disorder by the grading net. The shade of the cells corresponds to the prediction accuracy, with darker shades representing greater prediction accuracy for that category, i.e., a greater number of true positives. This visualisation allows us to pinpoint the categories where the network excels or encounters misclassification issues. By comparing the diagonal values (true positives) with the off-diagonal values (misclassifications), we can observe how accurately the network classifies and identify areas of frequent misclassification. Comparing the metrics in Table 7 with the confusion matrix in Figure 4 reveals that ICPNet outperforms the other models in classification accuracy, meeting essential requirements for disease recognition.

## 4. Discussion and Conclusions

### 4.1. Discussion

The effectiveness of ICPNet in recognising maize diseases was analysed and compared with GoogLeNet, ResNet-50, DenseNet-121, MobileNetV2, ResNeXt-50, RegNetY-400MF, CMT, and ICAI-V4. To better understand the recognition capabilities of the different networks, we used a confusion matrix. Figure 4 displays the confusion matrix for the classification outcomes of nine networks. In Table 7, it is evident that ICPNet outperforms the traditional mainstream networks in recognising the dataset.

GoogLeNet is able to capture multi-scale information within the same layer, and by combining convolutional filters of different sizes and pooling operations, the network processes and integrates information from a variety of receptive fields, thus improving the overall classification accuracy. Additionally, GoogLeNet effectively reduces computational costs through 1 x1 convolution. However, this complex Inception module structure makes the network more challenging to implement and optimise, and the deep architecture increases the risk of gradient vanishing, resulting in an average recognition accuracy of only 76.91%.

ResNet-50 addresses the concerns of declining learning performance and precision in order to increase web depth through its residual bloc design. The introduction of a shortcut in the remaining bloc allows information to be transferred between layers directly, preventing leakage and selective neglect in deep neural networks, thereby significantly improving the model’s recognition rate. This design ensures smooth data transmission within the network and effectively mitigates the problems of gradient vanishing and gradient explosion, enabling the construction of deeper network architectures. However, the average recognition accuracy remains at 78.60%.

DenseNet-121 utilises a densely interconnected architecture where the outlet of each layer serves as an input to all subsequent layers. This approach greatly decreases the number of parameters in the network and overcomes the challenges of vanishing gradients and sparseness of parameters. This design not only increases the module’s capability to generalise but also improves overall precision. Through this dense connectivity, DenseNet-121 achieves efficient reuse of features, allowing each layer to directly access the gradient information of the previous layers, further enhancing gradient transfer and feature propagation. Compared to traditional convolutional neural networks, this architecture reduces redundant computations, improves computational efficiency, and reduces the risk of overfitting. However, DenseNet-121 also has some shortcomings. Despite the reduction in the number of parameters, the network’s high-level, densely connected structure may still lead to a decrease in computational efficiency, and the average accuracy of its model is 79.38%.

MobileNetV2 optimises performance and reduces computational burden by using depthwise separable convolutions, drastically reducing the number of parameters and the amount of computation while maintaining network performance. This architecture significantly lowers computational costs and memory requirements by decomposing the default convolution into depth and point convolutions. MobileNetV2 also introduces an inverted residual structure and a linear bottleneck layer, further enhancing the expressiveness and efficiency of the model. However, MobileNetV2 also has some shortcomings. Despite its computational efficiency, the complexity of depthwise separable convolution may lead to increased difficulty in implementation and optimisation, and the average accuracy of its model is 81.25%.

ResNeXt-50 is an enhancement of ResNet-50 that further expands the net by implementing grouped convolution in the residual block. By learning different feature subspaces of the inputs, ResNeXt-50 improves the robustness of the network, achieving an average accuracy of 82.73%. Compared to ResNet-50, the network improved the average accuracy of identification of seven maize diseases by 4.13%. This enhancement greatly enhances the feature selection capability and classification performance of the module. However, the inclusion of the grouped convolution results in a substantial increase in the amount of parameters and a decrease in recognition efficiency. Additionally, more parameters and more complex computations imply higher memory requirements and longer training times. Nonetheless, ResNeXt-50 performs well in handling complex image classification tasks, particularly in improving the recognition accuracy of specific diseases.

RegNetY-400MF employs a rule-based network design methodology to construct the model by methodically optimizing the width and depth of each layer, targeting uniform and efficient performance. This strategy significantly reduces the number of parameters and computational requirements of the model by accurately controlling the number of channels and the recurrence of layers, effectively balancing performance and flexibility. The mean accuracy of the module is 84.75%.

RegNetY-400MF employs a rule-based network design methodology to construct models by methodically refining the width and depth of every layer, aiming for uniform and efficient performance. This strategy significantly reduces the number of parameters and computational requirements of the model by accurately controlling the number of channels and the repetition of layers, effectively balancing high efficiency and adaptability, with an average model accuracy of 84.75%. RegNetY-400MF excels in optimising the structure of the network and ensuring robust performance across a wide range of tasks through a rule-based design approach. However, despite the excellent performance of RegNetY-400MF in terms of parameters and computational requirements, this regularised design approach may have limitations when dealing with particularly complex tasks. Specifically, the regularised design may limit the flexibility of the model from performing optimally in some specific tasks. Additionally, despite the reduced number of parameters and computational requirements, the model may require further tuning and optimisation to maintain high performance when dealing with larger or more complex datasets.

The CMT network combines the advantages of Convolutional Neural Network (CNN) and Transformer techniques, enhancing the model’s capability to handle both local details and global dependencies by embedding the convolutional operation in the Transformer’s attention mechanism. The model achieves a mean accuracy of 85.37%. This fusion approach not only enhances the performance of the module to capture both detailed and global details in pictures but also demonstrates excellent performance in complex image classification tasks. However, despite the excellent performance of the CMT network in terms of accuracy and feature processing, this fusion architecture also imposes higher computational complexity and resource requirements. Specifically, the combination of convolutional operations and attention mechanisms increases the computational cost and memory footprint of the model, making it potentially challenging to apply in resource-constrained environments. Additionally, the complexity of the model increases the difficulty of implementation and optimisation, requiring more engineering skills.

ICAI-V4 introduces an enhanced Canny operator filtering technique to highlight edge features and reduce noise, and a coordinated attention scheme to improve feature detection and general model performance. This approach achieves an average model accuracy of 86.57%. This improvement allowed ICAI-V4 to excel in edge detection and feature extraction, significantly improving the accuracy of image classification. However, although ICAI-V4 performs well in edge feature extraction and noise minimisation, it does not address the problem of background interference. This means that in images containing complex backgrounds, the model may suffer from a certain degree of interference, which affects the classification results. Additionally, the combination of the improved Canny operator and the coordinated attention mechanism increases the computational complexity, leading to higher computational resource requirements and longer processing times.

Additionally, to validate the model’s capability in recognising different types of plant leaves, we utilised the public dataset PlantVillage for our tests. The results, presented in Table 8, show that ICPNet attained an average accuracy of 97.41%. The study outlines the reasons why ICPNet surpasses other mainstream networks.

1. In order to emphasise the tiny spot features of maize diseases, this study improved the cross-attention, channel, and spatial reconstruction modules to emphasise the tiny feature images of maize diseases. From the experimental results, the classification accuracy and precision of the maize disease model improved by 2.9% and 2.4%, respectively, after applying this method.

2. In order to effectively capture fuzzy edge features, a Coordinate Depthwise Convolution (CDC) is introduced in this paper. The experimental results show that the inclusion of CDC improves the classification accuracy of maize diseases by 3.2% and 2.6%, respectively.

3. In order to cope with model overfitting and stabilise the model training, we stabilised the model loss curve using the PLOA algorithm. As can be seen from the experimental data, these measures effectively improve the accuracy of maize disease classification.

4. As shown in the ablation experiments, the three models of our network performed different functions, including extracting tiny features, solving the edge blur problem, and stabilising model training. The integration of these models markedly improves all network performance and has a substantial positive impact on the module’s effectiveness.

5. Our study shows that this approach surpasses other deep neural network models in classification accuracy, achieving an average accuracy of 88.4%.

Overall, The ICPNet model excelled in classifying maize diseases and demonstrated strong performance in identifying various other plant leaf types. However, due to the diversity of maize diseases and the complexity of leaf characteristics, this study does not sufficiently consider the detection of other conditions and the similarities between different conditions. In addition, the present method focusses mainly on disease identification at the point of image acquisition and has not yet been extended to large-scale monitoring of the entire maize planting process and disease dynamics. In the future, in order to promote this method and achieve wider application, it is recommended to combine various technologies, such as the agricultural Internet of Things (IoT), to realise real-time monitoring of crop growth and pest and disease dynamics. This will not only enhance the modernisation of the agricultural sector but also drive its intelligent development.

### 4.2. Conclusions

In this paper, we introduce an innovative maize disease identification method called ICPNet. This method integrates multiscale convolution and gated convolution mechanisms and incorporates Integrated Multidimensional Attention (IMA) and Coordinate Depthwise Convolution (CDC) to enhance the model’s classification accuracy and robustness. Through a series of experimental validations, the method in this paper performs well in maize leaf images with complex backgrounds and diverse disease spots, significantly improving recognition accuracy. Specifically, the IMA module optimises the relationships and dependencies between the diseased spot elements by enhancing the expressiveness of the channel and spatial dimensions, significantly improving the generalisation of the model in practical applications. The CDC module effectively captures the disease features in different dimensions through the multiscale convolutional structure, enhancing the model’s accuracy and computational efficiency in dealing with fuzzy edge features. Additionally, the PSO-Integrated Lion Optimisation Algorithm (PLOA) is proposed to further improve the stability of model training and classification accuracy by adaptively adjusting the learning rate and optimisation parameters. The study results illustrate that the PLOA optimiser performs better in the maize disease classification task with complex features, proving its effectiveness and practicality.

Maize disease detection is a crucial technology for agricultural production and crop management, providing accurate information on crop health and helping farmers assess the type and severity of diseases. This technology helps farmers stay informed about field conditions and crop performance, enabling them to make more scientific management decisions. In this way, maize protection effectiveness and yields can be greatly improved while reducing losses from disease and promoting healthy and sustainable agricultural production. However, maize disease detection also faces challenges, such as the difficulty of feature extraction due to the high similarity between maize leaf disease species and the susceptibility of maize leaf images to noise during acquisition and transmission. To address these issues, this paper proposes ICPNet, which has achieved better results in maize disease detection.

The experimental results illustrate that our proposed ICPNet network has high accuracy and stability, but there is still some room for improvement in terms of parameter size and training time. Additionally, there are many types of maize diseases with different backgrounds. In future research, we will train the model on more disease types, different backgrounds, and mixed disease images to enhance its detection capabilities. We will continue to explore more advanced technologies to further develop the efficiency and precision of the module, thereby promoting the intelligent advancement of agriculture.

## Figures and Tables

**Table 1:** Special features of the data set on maize diseases.

Category	Example	Characteristics	Proportion
Blight	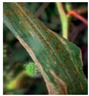	Fungal disease causes leaf and stem necrosis, leading to wilting and yield loss.	20.9%
Cercospora	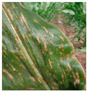	Fungal leaf spots expand in warm, humid conditions, causing premature leaf drop.	12.1%
Gray spot	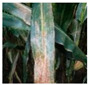	Fungal disease with grey lesions that disrupt photosynthesis and weaken plants.	21.2%
Fallarmy worm	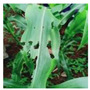	Damage from improper herbicide use, causing yellowing and necrosis of leaves.	10.3%
Herbicideburn	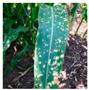	Herbicide burn in corn results from excessive chemical exposure, causing yellow or brown leaf damage.	9.6%
Rust	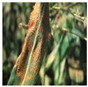	Rust in corn is a fungal disease that creates orange or brown spots on the leaves, potentially harming the plant’s health and yield.	10.6%
Zinc deficiency	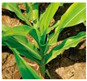	Nutrient disorder causing chlorosis and stunted growth, affecting younger leaves.	7.5%
Healthy Leaf	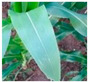	Leaves free of disease, pests, and deficiencies, showing uniform green colour and good growth.	7.8%

**Table 2:** Specific training parameter settings.

Specific Parameters	Value
Number of training images	3029
Number of test images	756
Input image size	256 × 256
Learning rate	1 × 10^−3^
Optimiser selection	PLOA
Weight decay factor	1 × 10^−2^
Learning rate adjustment strategies	Polynomial decay strategy
Batch_size	24
Training epochs	150

**Table 3:** Comparison of different attention mechanisms.

Method	Acc	Precision	Recall
Without attention	0.821	0.833	0.813
SE	0.811	0.813	0.802
CBAM	0.825	0.829	0.833
CA	0.841	0.844	0.839
ECA	0.823	0.811	0.806
TA	0.821	0.841	0.811
IMA	0.845	0.853	0.855

**Table 4:** Comparison of different convolutions.

Method	Acc	Precision	Recall
7 × 7 Conv	0.821	0.833	0.813
Ghost Conv	0.836	0.840	0.849
Involution	0.824	0.841	0.837
Atrous Conv	0.832	0.848	0.841
CDC	0.847	0.855	0.859

**Table 5:** Comparison of different optimisers.

Method	Acc	Precision	Recall
Adam	0.821	0.833	0.813
SGD	0.828	0.826	0.836
Adamw	0.823	0.825	0.839
Lion	0.835	0.836	0.844
PLOA	0.845	0.848	0.847

**Table 6:** Comparison of ablation results.

Resnext-50	IMA	CDC	PLOA	Acc	Precision
				0.821	0.833
	√			0.845	0.853
		√		0.847	0.855
			√	0.845	0.848
	√	√		0.857	0.858
	√		√	0.855	0.868
		√	√	0.866	0.870
	√	√	√	0.884	0.879

**Table 7:** Comparison of experimental results.

Methods	Precision	F1	Recall	Accuracy	FPS
GoogLeNet [35]	0.7612	0.7658	0.7705	0.7691	273.68
ResNet-50 [36]	0.7916	0.7828	0.7742	0.7860	246.59
DensNet-121 [37]	0.8035	0.7971	0.7908	0.7938	189.72
MobileNetV2 [38]	0.8155	0.8129	0.8103	0.8125	208.43
ResNeXt-50 [39]	0.8388	0.8310	0.8233	0.8273	230.85
RegNetY-400MF [40]	0.8460	0.8439	0.8418	0.8475	244.27
CMT [41]	0.8541	0.8525	0.8509	0.8537	233.32
ICAI-V4 [42]	0.8615	0.8634	0.8653	0.8657	246.71
ICPNet	0.8799	0.8767	0.8735	0.8846	285.56

**Table 8:** Generalisability experiments results.

Dataset	Accuracy (%)	Precision
PlantVillage grape	98.95	99.14
PlantVillage tomato	96.07	97.12
animals	97.20	97.46

## Data Availability

All the data obtained and materials analysed in this research are available from the corresponding author upon request.
